# Knockout of *TRDMT1* methyltransferase affects DNA methylome in glioblastoma cells

**DOI:** 10.1007/s11060-023-04304-8

**Published:** 2023-05-11

**Authors:** Tomasz Zabek, Tomasz Szmatola, Jagoda Adamczyk-Grochala, Anna Lewinska, Maciej Wnuk

**Affiliations:** 1 Department of Animal Molecular Biology, National Research Institute of Animal Production, Krakowska 1, 32-083 Balice, Poland; 2 Center for Experimental and Innovative Medicine, The University of Agriculture in Krakow, Redzina 1C, 30 248 Krakow, Poland; 3 Department of Biotechnology, Institute of Biology and Biotechnology, College of Nature Sciences, University of Rzeszow, Pigonia 1, 35-310 Rzeszow, Poland

**Keywords:** TRDMT1, DNA methylome, Reduced representation bisulfite sequencing (RRBS), Glioblastoma

## Abstract

**Purpose:**

We have previously shown that TRDMT1 methyltransferase is a regulator of chemotherapy-associated responses in glioblastoma cells. Despite the fact that glioblastoma, a common and malignant brain tumor, is widely characterized in terms of genetic and epigenetic markers, there are no data on TRDMT1-related changes in 5-methylcytosine pools in the genome. In the present study, the effect of *TRDMT1* gene knockout (KO) on DNA methylome was analyzed.

**Methods:**

CRISPR-based approach was used to obtain *TRDMT1* KO glioblastoma cells. Total 5-methylcytosine levels in DNA, DNMT1 pools and DNMT activity were studied using ELISA. Reduced representation bisulfite sequencing (RRBS) was considered to comprehensively evaluate DNA methylome in glioblastoma cells with *TRDMT1* KO.

**Results:**

*TRDMT1* KO cells were characterized by decreased levels of total 5-methylcytosine in DNA and DNMT1, and DNMT activity. RRBS-based methylome analysis revealed statistically significant differences in methylation-relevant DMS-linked genes in control cells compared to *TRDMT1* KO cells. *TRDMT1* KO-associated changes in DNA methylome may affect the activity of several processes and pathways such as telomere maintenance, cell cycle and longevity regulating pathway, proteostasis, DNA and RNA biology.

**Conclusions:**

TRDMT1 may be suggested as a novel modulator of gene expression by changes in DNA methylome that may affect cancer cell fates during chemotherapy. We postulate that the levels and mutation status of *TRDMT1* should be considered as a prognostic marker and carefully monitored during glioblastoma progression.

**Supplementary Information:**

The online version contains supplementary material available at 10.1007/s11060-023-04304-8.

## Introduction

Glioblastoma is a common and aggressive brain tumor. Although a number of anti-glioblastoma therapies are available (e.g., surgery, radiotherapy and chemotherapy), prognosis is poor and long-term survival is limited [1]. Glioblastoma is characterized by spatial and temporal heterogeneity at genomic, transcriptomic and epigenomic levels that may contribute to drug resistance and cancer recurrence [2–7]. However, the interdependence of these aberrations/alterations is not fully understood. For example, mutation of a single gene, isocitrate dehydrogenase 1 (*IDH1*) is a driver of the CpG island methylator phenotype (CIMP) in gliomas [8]. The lack of functional *IDH1* gene in primary human astrocytes promoted extensive DNA hypermethylation and reshaped the methylome that mimicked the changes noted in G-CIMP-positive lower-grade gliomas [8]. More studies are needed to highlight the interplay between genetic and epigenomic changes in glioblastoma.

Despite the fact that TRDMT1 methyltransferase was initially classified as DNA methyltransferase (former name: DNMT2), TRDMT1-mediated DNA methylating activity is established to be residual [9]. In contrast, TRDMT1 is documented to be a 5-methylcytosine RNA methyltransferase involved in cellular stress responses such as oxidative stress and DNA damage response [10–13]. *TRDMT1* knockout (KO) increased sensitivity to PARP inhibitors in osteosarcoma cells and TRDMT1 was proposed to be a novel target in DNA damage-based anti-cancer therapies [13]. More recently, we have shown that *TRDMT1* KO may modulate chemotherapy-associated responses that affected genetic stability and promoted cellular heterogeneity in stress-induced senescent glioblastoma cells [14]. *TRDMT1* KO also affected microRNA pools during doxorubicin-stimulated ER stress in glioblastoma cells [15]. However, there are no data on TRDMT1-mediated changes in DNA methylome and related signaling pathways in cancer cells.

In the present study, the effect of CRISPR-based *TRDMT1* KO on DNA methylation at a genome-wide scale at single-nucleotide resolution was analyzed using a cellular model of glioblastoma in vitro, namely U-251 MG cell line. Reduced representation bisulfite sequencing (RRBS) revealed significant differences in methylation-relevant DMS-linked genes in *TRDMT1* KO glioblastoma cells. Signaling pathways associated with DMS-linked genes were also identified by the means of gene ontology (GO) analysis.

## Materials and methods

### Cell lines, growth conditions and gene knockout

U-251 MG human glioblastoma cell line was used (09063001, ECACC, Public Health England, Porton Down, Salisbury, UK). Cells were routinely cultured as described elsewhere [14]. CRISPR-based approach was used to knockout the *DNMT2*/*TRDMT1* gene as previously described [14]. Briefly, cells were transfected with DNMT2 Double Nickase Plasmids (Santa Cruz Biotechnology, Dallas, TX, USA) using Lipofectamine^™^ 3000 (Thermo Fisher Scientific, Waltham, MA, USA) according to the manufacturer’s instructions. Plasmid-mediated effects (control cells, C-NIC cells) were also monitored using Control Double Nickase Plasmid (Santa Cruz Biotechnology). Cells with *DNMT2/TRDMT1* KO (D-NIC cells) were selected in the presence of puromycin (sc-108071, Santa Cruz Biotechnology, Dallas, TX, USA) and *DNMT2/TRDMT1* gene knockout was confirmed using anti-DNMT2 antibody (sc-271513, Santa Cruz Biotechnology) and western blotting [14].

### ELISA-based analysis of total DNA methylation

DNA was isolated using a GenElute™ Mammalian Genomic DNA Miniprep Kit (Merck KGaA, Darmstadt, Germany). The levels of 5-methylcytosine in DNA samples (100 ng) were evaluated using MethylFlash™ Global DNA Methylation (5-mC) ELISA Easy Kit (EpiGentek, Farmingdale, New York, USA) and an absorbance microplate reader according to the manufacturer’s instruction. Results were normalized to C-NIC cells.

### ELISA-based analysis of DNMT1 levels and DNMT activity

The nuclear extracts were obtained as previously described [16]. DNMT1 (DNA methyltransferase) quantification and DNMT activity assays were performed using 5 μg of nuclear extract, an EpiQuik™ DNMT1 Assay Kit and an EpiQuik^™^ DNA Methyltransferase Activity/Inhibition Assay Kit (EpiGentek), respectively, according to the manufacturer’s instruction.

### Construction of reduced representation bisulfite sequencing (RRBS) library

DNA was isolated from C-NIC and D-NIC glioblastoma cells and quantified using QUBIT fluorometer (Broad Range dsDNA Assay, Thermo Fisher Scientific). RRBS library was created using normalized DNA samples and Premium RRBS kit (Diagenode, Ougrée, Belgium). 100 ng of DNA were digested with methyl-insensitive restriction enzyme (MspI) to obtain DNA fragments containing at least one CpG site. Digested DNA samples were ligated to methylated adapters including indexes and were purified using magnetic beads (AMPure XP Reagent, Beckman Coulter, Brea, USA). DNA was then bisulfite converted and enriched using PCR amplification. Obtained RRBS libraries were quantified using QUBIT High Sensitivity dsDNA Assay (Thermo Fisher Scientific) and their quality was assessed using High Sensitivity DNA ScreenTape Analysis of the TapeStation system (Agilent Technologies, Santa Clara, CA, USA). Three pools of four indexed equimolar RRBS libraries were subjected to next generation sequencing (NGS) on the Hiseq Illumina system using 150 cycles of pair end mode of NGS.

### Bioinformatics analysis

The raw reads were checked for quality assessment with the use of FastQC software (http://www.bioinformatics.babraham.ac.uk/projects/fastqc), followed by trimming procedure that allowed to remove reads that did not fulfill the following criteria: < 20 phred quality, reads shorter than 35 bp, presence of adapters (Flexbar) [17]. After trimming procedure, the filtered reads were mapped to human HG38 genome with the use of BSMAP software set for RRBS analysis [18], followed by transformation of the result files to bed files used as RnBeads input [19]. Next, the RnBeads analysis was performed using Bmiq normalization method with human HG38 reference genome selected. For the visualization of RRBS results, analytics platform WeiShengxin (http://www.bioinformatics.com.cn) was used. The RRBS results were presented as peak chromosome distribution and circle plot. Enriched horizontal bars with colors were created using WeiShengxin on the basis of data generated with DIANA tools.

### Annotation of identified DM CpG sites and gene overrepresentation tests

The annotation of variably methylated CpG sites was provided using reference human genome (build HG38) using variant effect predictor tool of the Ensembl database (version 104). In order to retrieve biological context of observed differential methylation, overrepresentation tests were performed for genes linked to differentially methylated CpG sites using functional annotation chart of DAVID web available tools (https://david.ncifcrf.gov/). The data collected from the Functional Annotation search included gene groups identified in a specific biological process (GOTERM BP DIRECT), molecular function (GOTERM MF DIRECT), cellular compartment (GOTERM CC DIRECT), Uniprot keywords (UP KEYWORDS), and KEGG pathway term (KEGG PATHWAY). Functional Annotation Clustering uses an algorithm to reduce redundant term association and provides an enhanced biological interpretation of specific gene groups being analyzed. For the visualization of DMS results, analytics platform WeiShengxin (http://www.bioinformatics.com.cn) was used. The DMS results were presented as Gene Ontology (GO), chord and enrichment bubble plot.

### Statistical analysis

ELISA-based results are presented as the mean ± SD from at least three independent experiments. Differences between C-NIC cells and D-NIC cells were evaluated using Student’s *t*-test. Statistical significance was analyzed using GraphPad Prism 5. *P*-values of less than 0.05 were considered significant.

## Results and discussion

### *TRDMT1* KO results in decreased levels of total 5-mC in DNA, DNMT1 levels and activity in glioblastoma cells

Data on TRDMT1-mediated effects on human methylome and related signaling pathways are limited [20]. RNA sequencing (RNA-Seq) and RNA bisulfite sequencing (RNA-BisSeq) were used to correlate the differentially methylated genes with differentially expressed genes (DEGs) in human embryonic kidney cell line HEK293 without active *TRDMT1* gene [20]. The authors showed that differentially expressed genes were associated with the cell cycle, RNA transport, and RNA degradation and were enriched in cancer and Notch signaling pathways [20]. Thus, *TRDMT1* KO affected RNA methylome that was accompanied by limited proliferation and migration of HEK293 cells [20]. However, the authors did not consider TRDMT1-mediated effects on RNA methylome and associated changes in the activity of signaling pathways in cancer cells. To the best of our knowledge, there are also no data on TRDMT1-based modulation of DNA methylome.

As TRDMT1 may regulate cellular stress responses by 5-methylcytosine modification of RNA [13, 21], we were also interested if the lack of functional *TRDMT1* gene may affect DNA methylome landscape in cancer cells. We have selected U-251 MG glioblastoma cells for the current analysis for several reasons. *TRDMT1* KO in glioblastoma cells resulted in elevated DNA damage and impaired RNA-mediated DNA damage response (DDR) that promoted cellular heterogeneity during drug-induced senescence [14]. *TRDMT1* KO also affected the levels of 5-methylcytosine RNA methyltransferase NSUN family in chemotherapy-stimulated senescent glioblastoma cells [14]. Furthermore, *TRDMT1* KO modulated microRNA pools during doxorubicin-induced ER stress in glioblastoma cells [15]. Thus, TRDMT1 may regulate gene expression by different mechanisms in glioblastoma cells.

**Fig. 1:**
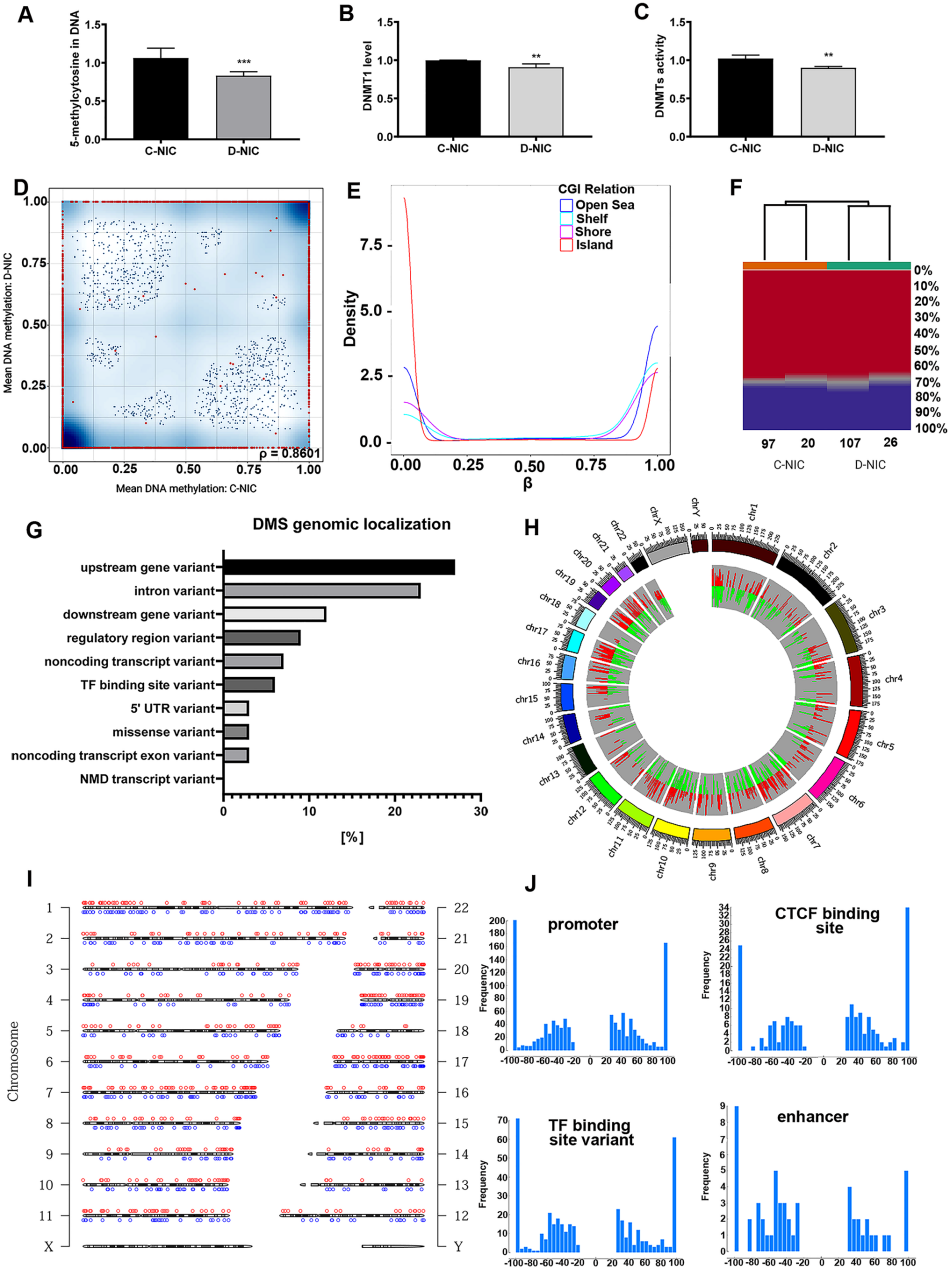
The effect of *TRDMT1* KO on DNA methylation-related parameters in U-251 MG cells. **A** ELISA-based analysis of total 5-methylcytosine (5-mC) content in gDNA. Results were normalized to control cells (C-NIC cells), n = 6, *p* < 0.001 compared to C-NIC cells (Student’s t-test). **B** ELISA-based analysis of DNMT1 levels in nuclear extract. Results were normalized to control cells (C-NIC cells), n = 6, *p* < 0.01 compared to C-NIC cells (Student’s t-test). **C** ELISA-based analysis of DNMT activity in nuclear extract. Results were normalized to control cells (C-NIC cells), n = 6, *p* < 0.01 compared to C-NIC cells (Student’s t-test). **D** Differential methylation levels in the CpG sites in C-NIC versus D-NIC cells. **E** Distribution of DNA methylation (beta values) in terms of variable CpG content in the human genome of C-NIC versus D-NIC cells. **F** Methylome clustering using a heat map based on differential methylation in C-NIC (97 and 20), and D-NIC (107 and 26) cells. Y-axis (right) of each cluster includes the percentage of CpG methylation (%). **G** Genomic localization of differentially methylated CpG sites in C-NIC and D-NIC cells. **H** Circle plot with the distribution of hypermethylation- (red) and hypomethylation-related (green) changes in human somatic chromosomes using WeiShengxin software (http://www.bioinformatics.com.cn). **I** Chromosome ideogram plot presenting the distribution of hypermethylated (red) and hypomethylated (blue) sites in human somatic chromosomes using WeiShengxin software (http://www.bioinformatics.com.cn). **J** Histograms showing the frequency (Y-axis) of differential methylation (X-axis) at CpG sites in the human genome covering promoters, TF binding sites, CTCF binding sites and enhancers in C-NIC (left) and D-NIC cells (right). The hypomethylation in one group is presented as the differential methylation values ranging from -100 (the lack of methylation in the first group versus the second group) to -25 (upper threshold of hypomethylation in the first group versus the second one). The hypermethylation in one group is presented as the differential methylation values ranging from 25 (lower threshold of hypermethylation in the first group versus the second one) to 100 (complete methylation in the first group versus the second one)

First, total DNA methylation in *TRDMT1* KO glioblastoma cells was initially evaluated using ELISA-based approach (Fig. 1A). *TRDMT1* KO resulted in a decrease in global DNA methylation of about 15% compared to control cells with unaffected levels of TRDMT1 (Fig. 1A). This observation was accompanied by decreased levels of DNMT1 (Fig. 1B) and DNMT activity (Fig. 1C). This is not surprising as DNMT1 is the major enzyme responsible for maintaining DNA methylation patterns following DNA replication. We have previously documented that siRNA-based silencing of *TRDMT1* stimulated changes in global DNA methylation and the levels of DNMTs in normal human fibroblasts [12].

### *TRDMT1* KO modulates DNA methylome in glioblastoma cells

Second, reduced representation bisulfite sequencing (RRBS) was used to provide a comprehensive analysis of differentially methylated sites in *TRDMT1* KO glioblastoma cells (Fig. 1D–J). Indeed, *TRDMT1* KO resulted in affected DNA methylome in glioblastoma cells as judged by pairwise CpG methylation differences between control cells (C-NIC cells) and *TRDMT1* KO cells (D-NIC cells) (Fig. 1D–J). Using RRBS pair-end sequencing, we obtained about 28 million of sequencing reads per sample from which 57% were uniquely aligned to the human reference genome. The scatterplots of mean differential methylation in pairwise comparisons between C-NIC and D-NIC cells were generated based on 41,240 CpGs (false discovery rate < 0.05) (Fig. 1D). The analysis of comparative global methylation levels revealed the high correlation coefficient in CpG sites between C-NIC and D-NIC cells (ρ value of 0.8601, Fig. 1D). The global distribution of DNA methylation was found to be bimodal in C-NIC versus D-NIC cells (Fig. 1E). The majority of CpG sites between C-NIC and D-NIC cells were characterized by either high or low methylation (distribution of β values, Fig. 1E) independent on CpG structures found in the human genome. CpG structures include CpG islands (CGI), which are flanked by shores (up to 2 kb) and shelves (2–4 kb from CGI) and also CpGs found in the open seas, which represent the rest parts of the genome [22]. Regardless of *TRDMT1* KO, the biggest number of hypomethylated CpGs were found in CpG islands, and the biggest number of hypermethylated CpGs were observed in open seas (Fig. 1E). Hierarchical clustering showed significant differences in methylation between C-NIC and D-NIC cells (Fig. 1F). 2490 DMS between C-NIC and D-NIC cells (false discovery rate below 0.05 of differential methylation values) were selected for further analysis. The annotation of significant DM CpG sites in the human genome (Table S1) showed that the majority of them were located in upstream gene regions and in intronic sequences (Fig. 1G). The other ones, included CpG sites, were located downstream to gene boundaries, in regulatory regions, transcription factor binding sites, 5 prime UTR regions, and coding sequences (Fig. 1G). Alignment to the reference sequence of the human genome revealed 1529 DMS between C-NIC and D-NIC cells localized in loci of 2828 genes and 1588 regulatory features (Table S1). Comparative analysis of DNA methylome showed that 751 sites were hypermethylated and 778 sites were hypomethylated in C-NIC cells compared to D-NIC cells (Fig. 1H). The following hypermethylated and hypomethylated regions were noted on particular chromosomes, namely chr 1 (69 and 96), chr 2 (43 and 52), chr 3 (28 and 38), chr 4 (27 and 28), chr 5 (35 and 35), chr 6 (37 and 22), chr 7 (82 and 73), chr 8 (31 and 43), chr 9 (31 and 28), chr 10 (42 and 46), chr 11 (41 and 37), chr 12 (18 and 22), chr 13 (10 and 15), chr 14 (19 and 19), chr 15 (26 and 23), chr 16 (23 and 29), chr 17 (69 and 57), chr 18 (6 and 14), chr 19 (53 and 45), chr 20 (39 and 35), chr 21 (9 and 6), chr 22 (13 and 15), respectively (Fig. 1I). According to the Ensembl annotation of regulatory elements of genes, the highest number of differentially methylated CpG sites was located in promoters, which were directly associated with the occurrence of coding loci (Fig. 1J). Some others were also commonly found in TF binding- and CTCF binding sites (Fig. 1J). Some DM CpGs also covered the enhancers (Fig. 1J). The lack of DM CpG sites showing differential methylation values in the range above -25 and below 25 resulted from of applied cut off values of differential methylation in this study (Fig. 1J). Observed pattern of the mean values of differential methylation in case of mentioned regulatory elements was characterized by bimodal distribution except to differentially methylated CpGs covering the enhancers (Fig. 1J).

### Characterization of DMS linked genes involved in general methylation-relevant processes and telomere biology in *TRDMT1* KO cells

*TRDMT1* KO in glioblastoma cells resulted in the presence of DM loci of genes that products methylate DNA or histone proteins (Table 1). Functional annotation using DAVID tools also showed the presence of 15 DMS linked genes involved in telomere biology such as telomerase activity, telomere assembly, a number of mechanisms of telomere maintenance and protection (Table S2). This group comprised the following hypomethylated genes, namely *WRN*, *UPF1*, *ACD*, *ERCC1*, *HNRNPA2B1*, *HSP90AA1*, *TERC, CERS1* and hypermethylated genes, namely *RUVBL2*, *MAP2K7*, *CDC45* in C-NIC cells compared to D-NIC cells (Table S2). In addition, aforementioned DMS linked genes may be implicated in the regulation of cell cycle, cellular senescence and cellular responses to DNA damage (Table S2).

**Table 1:** Functional annotation results of methylation-relevant DMS-linked genes between C-NIC and D-NIC cells

GO Biological process	DMS-linked gene (C-NIC vs D-NIC cells)
GO:0080111 ~ DNA demethylation	*EOMES*
GO:0006306 ~ DNA methylation	*EOMES* *HEMK1* *MGMT*
GO:0043046 ~ DNA methylation involved in gamete generation	*MAEL*
GO:0051568 ~ histone H3-K4 methylation	*NCOA6*
GO:0034972 ~ histone H3-R26 methylation	*PRDM14*
GO:0016571 ~ histone methylation	*PRDM6*
GO:0032259 ~ methylation	*METTL21A* *MGMT* *PRDM14*
GO:1901536 ~ negative regulation of DNA demethylation	*GATA3*
GO:0018022 ~ peptidyl-lysine methylation	*METTL21A*
GO:1905643 ~ positive regulation of DNA methylation	*WT1*
GO:0051571 ~ positive regulation of histone H3-K4 methylation	*AUTS2*
GO:1900111 ~ positive regulation of histone H3-K9 dimethylation	*PRDM12*
GO:0006479 ~ protein methylation	*HEMK1* *METTL21A*
GO:0044030 ~ regulation of DNA methylation	*PRDM14*
GO:0061085 ~ regulation of histone H3-K27 methylation	*GATA3*

### DMS linked gene overrepresentation analysis in GO terms based on gene functional annotation chart in *TRDMT1* KO cells

**Fig. 2:**
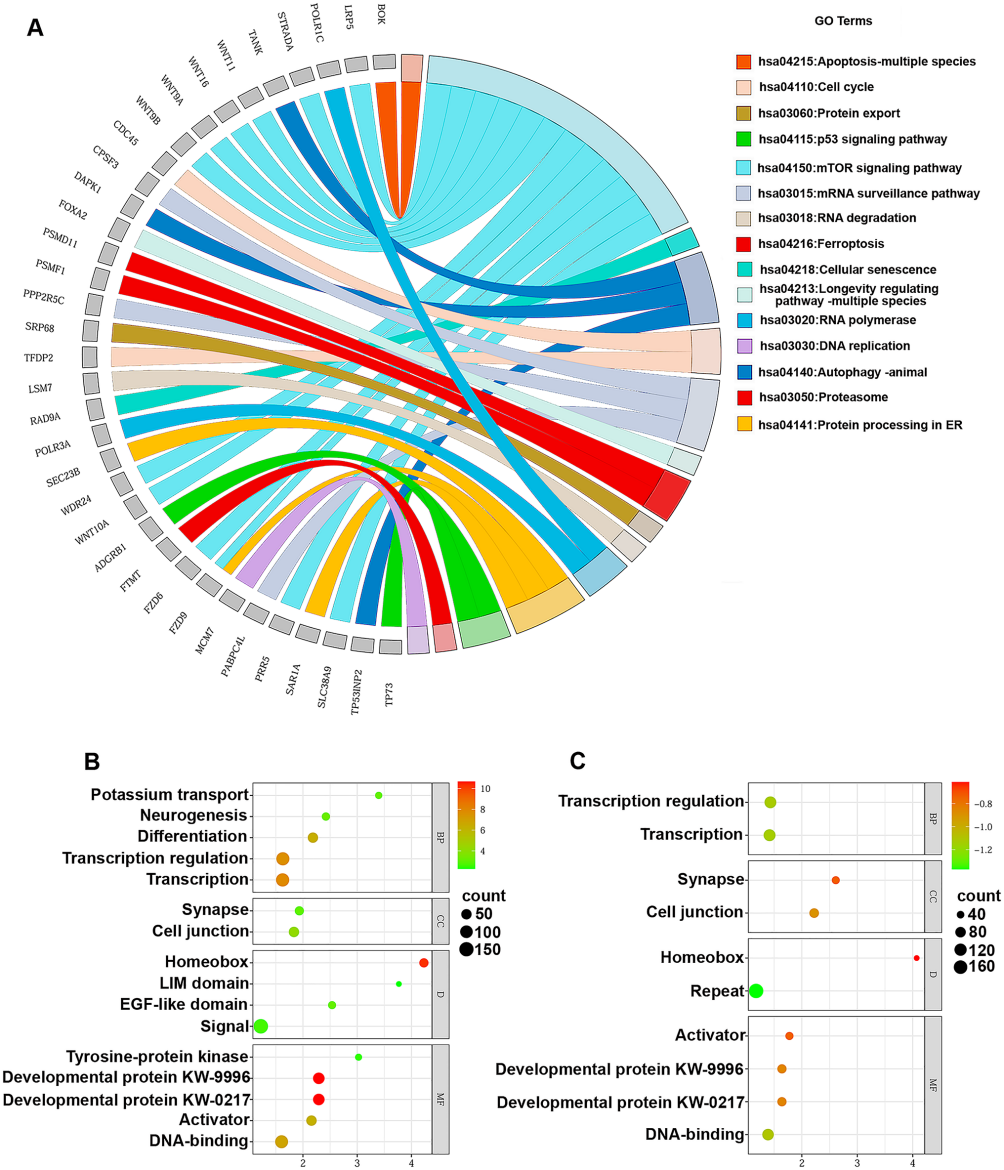
Analysis of overrepresentation results of DMS linked genes in GO terms in C-NIC versus D-NIC cells. DMS results are presented as GO chord (**A**) and enrichment bubble plots (**B**, **C**). The results of overrepresentation in GO terms for genes covering enhancer-related CpG sites being both hypo- or hypermethylated in C-NIC versus D-NIC cells were obtained using functional annotation chart of DAVID web available software. Uniprot Keywords (UP KW) were considered. **A** 35 enhancer-DMS linked genes that are crucial for cellular process are shown. The analysis was based on 15 GO terms. **B** The overrepresented genes hypermethylated in C-NIC versus D-NIC cells. **C** The overrepresented genes hypomethylated in C-NIC versus D-NIC cells. C-NIC cells, control cells; D-NIC cells, cells with *TRDMT1* KO; BP, biological process; CC, cellular component; D, domain; MF, molecular function

Overrepresentation analysis in GO terms using DAVID tools revealed a list of processes being potentially affected in *TRDMT1* KO cells that are relevant to the functioning of nervous system such as neuron differentiation based on the group of hypomethylated and hypermethylated genes in C-NIC versus D-NIC cells (Table S3). 36 DMS linked genes were selected for additional analysis of 15 GO terms crucial for cellular process such as apoptosis, mTOR signaling pathway, cellular senescence, autophagy, cell cycle, mRNA surveillance pathway, longevity regulating pathway, proteasome, protein export, RNA degradation, RNA polymerase, protein processing in endoplasmic reticulum, p53 signaling pathway, ferroptosis and DNA replication. We have found that the most DMS linked genes were involved in mTOR signaling pathway (Fig. 2A). A detailed information is also provided in Table S3. Based on DAVID tool analysis, hypermethylated genes classified to 16 pathways were revealed in C-NIC vs D-NIC cells (Fig. 2B). The most affected genes were found in UP KW categories of domain (signal), biological processes (transcription and transcription regulation) and molecular function (developmental proteins) (Fig. 2B). Furthermore, hypomethylated genes grouped to 10 pathways were observed in C-NIC versus D-NIC cells (Fig. 2C). The most affected genes were noticed in UP KW categories of domain (homeobox) and cellular component (cell junction, synapse) (Fig. 2C).

### Overrepresentation results of enhancer-DMS linked genes in GO terms

Due to irregular distribution of differential methylation values of enhancer-related CpG sites between C-NIC and D-NIC cells, we have also performed overrepresentation testing for their co-occurred genes. As a result, we found a list of 47 genes significantly overrepresented in selected GO terms (*p* < 0.05) (Table S4). DAVID overrepresentation test showed that enhancer-related CpG sites variably methylated between C-NIC and D-NIC cells were found in loci of genes encoding transcription factors (mostly homeobox proteins) overrepresented in DNA binding processes (Table S4).

As we have already stated, there are no data on *TRDMT1* KO-mediated effects on DNA methylome in cancer cells. However, there were attempts to analyze the consequences of *TRDMT1* KO in human normal cells (HEK293 cells) in terms of RNA methylome and associated DEGs [20]. The authors concluded that TRDMT1 is a regulator of mRNA methylation that limited proliferation and migration of HEK293 cells [20].

In conclusion, we have shown that apart from TRDMT1-mediated regulation of gene expression by RNA modification [20], microRNA [15] and lcnRNA [23], TRDMT1 may be also a modulator of DNA methylation-based epigenetic control of gene expression. However, more studies are needed to reveal a molecular mechanism of TRDMT1-associated modulation of DNA methylome and its consequences in cancer cells. We propose that the levels and mutation status of *TRDMT1* should be analyzed during glioblastoma progression as novel prognostic markers.

## Supplementary Information

Below is the link to the electronic supplementary material.

Supplementary file1 (XLSX 2120 kb)

## Data Availability

The data presented in this study are available in the supplementary material.

## References

[1] Wen PY, Kesari S (2008). Malignant gliomas in adults. N Engl J Med.

[2] Patel AP, Tirosh I, Trombetta JJ (2014). Single-cell RNA-seq highlights intratumoral heterogeneity in primary glioblastoma. Science.

[3] Meyer M, Reimand J, Lan X (2015). Single cell-derived clonal analysis of human glioblastoma links functional and genomic heterogeneity. Proc Natl Acad Sci.

[4] Wang J, Cazzato E, Ladewig E (2016). Clonal evolution of glioblastoma under therapy. Nat Genet.

[5] Ceccarelli M, Barthel FP, Malta TM (2016). Molecular profiling reveals biologically discrete subsets and pathways of progression in diffuse glioma. Cell.

[6] Lee J-K, Wang J, Sa JK (2017). Spatiotemporal genomic architecture informs precision oncology in glioblastoma. Nat Genet.

[7] Klughammer J, Kiesel B, Roetzer T (2018). The DNA methylation landscape of glioblastoma disease progression shows extensive heterogeneity in time and space. Nat Med.

[8] Turcan S, Rohle D, Goenka A (2012). *IDH1* mutation is sufficient to establish the glioma hypermethylator phenotype. Nature.

[9] Hermann A, Schmitt S, Jeltsch A (2003). The human Dnmt2 has residual DNA-(Cytosine-C5) methyltransferase activity. J Biol Chem.

[10] Goll MG, Kirpekar F, Maggert KA (2006). Methylation of tRNA ^Asp^ by the DNA methyltransferase homolog Dnmt2. Science.

[11] Lewinska A, Adamczyk-Grochala J, Kwasniewicz E, Wnuk M (2017). Downregulation of methyltransferase Dnmt2 results in condition-dependent telomere shortening and senescence or apoptosis in mouse fibroblasts. J Cell Physiol.

[12] Lewinska A, Adamczyk-Grochala J, Kwasniewicz E (2018). Reduced levels of methyltransferase DNMT2 sensitize human fibroblasts to oxidative stress and DNA damage that is accompanied by changes in proliferation-related miRNA expression. Redox Biol.

[13] Chen H, Yang H, Zhu X (2020). m5C modification of mRNA serves a DNA damage code to promote homologous recombination. Nat Commun.

[14] Bloniarz D, Adamczyk-Grochala J, Lewinska A, Wnuk M (2021). The lack of functional *DNMT2/TRDMT1* gene modulates cancer cell responses during drug-induced senescence. Aging.

[15] Adamczyk-Grochala J, Bloniarz D, Zielinska K (2022). *DNMT2/TRDMT1* gene knockout compromises doxorubicin-induced unfolded protein response and sensitizes cancer cells to ER stress-induced apoptosis. Apoptosis.

[16] Lewinska A, Wnuk M, Grabowska W (2015). Curcumin induces oxidation-dependent cell cycle arrest mediated by SIRT7 inhibition of rDNA transcription in human aortic smooth muscle cells. Toxicol Lett.

[17] Dodt M, Roehr J, Ahmed R, Dieterich C (2012). FLEXBAR—flexible barcode and adapter processing for next-generation sequencing platforms. Biology.

[18] Xi Y, Li W (2009). BSMAP: whole genome bisulfite sequence MAPping program. BMC Bioinformatics.

[19] Müller F, Scherer M, Assenov Y (2019). RnBeads 2.0: comprehensive analysis of DNA methylation data. Genome Biol.

[20] Xue S, Xu H, Sun Z (2019). Depletion of TRDMT1 affects 5-methylcytosine modification of mRNA and inhibits HEK293 cell proliferation and migration. Biochem Biophys Res Commun.

[21] Schaefer M, Pollex T, Hanna K (2010). RNA methylation by Dnmt2 protects transfer RNAs against stress-induced cleavage. Genes Dev.

[22] Schneider E, Dittrich M, Böck J (2016). CpG sites with continuously increasing or decreasing methylation from early to late human fetal brain development. Gene.

[23] Betlej G, Lewińska A, Adamczyk-Grochala J (2022). Deficiency of TRDMT1 impairs exogenous RNA-based response and promotes retrotransposon activity during long-term culture of osteosarcoma cells. Toxicol In Vitro.

